# Geometric dose prediction model for hemithoracic intensity‐modulated radiation therapy in mesothelioma patients with two intact lungs

**DOI:** 10.1120/jacmp.v17i3.6199

**Published:** 2016-05-08

**Authors:** LiCheng Kuo, Ellen D. Yorke, Vishruta A. Dumane, Amanda Foster, Zhigang Zhang, James G. Mechalakos, Abraham J. Wu, Kenneth E. Rosenzweig, Andreas Rimner

**Affiliations:** ^1^ Department of Medical Physics Memorial Sloan Kettering Cancer Center New York NY USA; ^2^ Department of Radiation Oncology Mount Sinai Medical Center New York NY USA; ^3^ Department of Radiation Oncology Memorial Sloan Kettering Cancer Center New York NY USA; ^4^ Department of Epidemiology and Biostatistics Memorial Sloan Kettering Cancer Center New York NY USA

**Keywords:** malignant pleural mesothelioma (MPM), intensity‐modulated radiation therapy (IMRT), dose prediction model

## Abstract

The presence of two intact lungs makes it challenging to reach a tumoricidal dose with hemithoracic pleural intensity‐modulated radiation therapy (IMRT) in patients with malignant pleural mesothelioma (MPM) who underwent pleurectomy/decortications or have unresectable disease. We developed an anatomy‐based model to predict attainable prescription dose before starting optimization. Fifty‐six clinically delivered IMRT plans were analyzed regarding correlation of prescription dose and individual and total lung volumes, planning target volume (PTV), ipsilateral normal lung volume and ratios: contralateral/ipsilateral lung (CIVR); contralateral lung/PTV (CPVR); ipsilateral lung /PTV (IPVR); ipsilateral normal lung /total lung (INTLVR); ipsilateral normal lung/PTV (INLPVR). Spearman's rank correlation and Fisher's exact test were used. Correlation between mean ipsilateral lung dose (MILD) and these volume ratios and between prescription dose and single lung mean doses were studied. The prediction models were validated in 23 subsequent MPM patients. CIVR showed the strongest correlation with dose (R=0.603,p<0.001) and accurately predicted prescription dose in the validation cases. INLPVR and MILD as well as MILD and prescription dose were significantly correlated (R=‐0.784,p<0.001 and R=0.554,p<0.001, respectively) in the training and validation cases. Parameters obtainable directly from planning scan anatomy predict achievable prescription doses for hemithoracic IMRT treatment of MPM patients with two intact lungs.

PACS number(s): 87.55.de, 87.55.dk

## I. INTRODUCTION

Intensity‐modulated radiation therapy (IMRT) to the entire hemithoracic pleura is a promising new strategy for patients with unresectable malignant pleural mesothelioma (MPM) or after pleurectomy/decortication.^1^, ^2^ However, it is a complex treatment to plan and deliver, and several publications^3^, ^4^, ^5^, ^6^ document the potential for serious radiation‐induced lung toxicity. We previously reported an unacceptably high rate of radiation pneumonitis after conventional radiation therapy to MPM patients with two intact lungs.^(3)^ We therefore developed a novel IMRT technique targeting the entire hemithoracic pleura. Lung radiation tolerance is the main limitation in planning these complex treatments, though other nearby normal tissues must also be protected. In our experience, the prescription goal of 50.4 Gy in 28 fractions is achieved in approximately 25% of patients, while another quarter can receive prescription dose of 48.6 Gy without exceeding normal tissue constraints.

Planning these treatments is a time‐consuming iterative cycle of optimization, plan evaluation, and reoptimization. Before the start of optimization, several quantities are available: the planning computerized tomography (CT) scan, delineated target and organ‐at‐risk (OAR) volumes, the dose constraints, and the desired prescription dose. It would be helpful to identify the highest prescription ≤50.4Gy) that meets OAR constraints from anatomical parameters among these quantities before beginning optimization. Therefore we analyzed the clinically delivered IMRT treatment plans from our institution and developed an anatomy‐based predictive model to allow such estimation. As a further aid to planners, we developed a model based on the correlation between anatomical variables and mean total and ipsilateral lung doses (MLD, MILD). The mean lung doses are often optimization constraints which can be evaluated and changed during the optimization. Knowing what to expect helps the planner steer the optimization toward the highest allowed prescription dose.

## II. MATERIALS AND METHODS

### A. Study design and patients

We reviewed treatment plans of all 56 MPM patients with unresectable or pleurectomy/decortication treated with definitive or adjuvant hemithoracic pleural IMRT at our institution between 2005 and 2012. An Institutional Review Board/Privacy Board waiver was approved prior to conducting this study. All patients were positioned supine with their arms over the head and immobilized in a customized Alpha Cradle mold (Smithers Medical Products, Inc., North Canton, OH). The target delineation technique has been previously described.^(6)^ Briefly, a planning CT scan was acquired with a slice thickness of 2.5‐3 mm. Since 2008, a respiratory‐correlated CT (RCCT) scan acquired at simulation was used to create an ITV. A recent positron emission tomography scan also aided the delineation of any potential gross disease. Typically the PTV extended from the thoracic inlet superiorly to the bottom of the L2 vertebral body inferiorly. It included all visualized gross disease and the CTV with an approximately 10 mm outer margin and a 6 mm inner margin with adjustments to accommodate respiratory motion. This resulted in an approximately 16 mm thick PTV rind completely surrounding the ipsilateral lung.^(6)^

**Figure 1:**
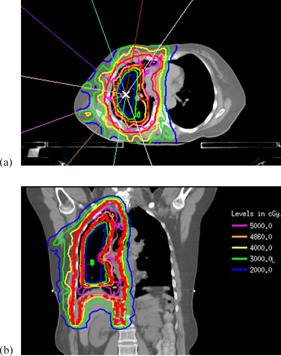
Typical hemithoracic pleural IMRT plan for MPM. PTV is represented by thick red lines: (a) beam arrangements and isodose distribution on axial view, (b) isodose distribution on coronal view.

The clinical treatment plans for the training set and the first 10 validation cases were performed on a previously described in‐house treatment planning system^(7)^ in which a radiological path‐length‐corrected, pencil‐beam algorithm accounted for tissue inhomogeneity. The later 13 validation cases were planned with the Eclipse v.11.0 treatment planning system (Varian Medical Systems, Palo Alto, CA) using the AAA (anisotropic analytical algorithm) dose calculation algorithm. All patients were treated with coplanar 6 MV photon beams using six to nine beam angles, approximately equispaced between 200° and 240° to encompass the ipsilateral lung. A sliding‐window IMRT technique^(8)^ on Varian linear accelerators (Varian Medical Systems) was used. Department guidelines regarding beam directions, optimization starting points, and target coverage and normal tissue planning goals were applied for all cases. Figure 1 shows axial and coronal views of a typical PTV, beam arrangement, and absolute dose distribution.

Ideally, we aim to deliver 50.4 Gy in 28 fractions, with prescription covering ≥95% of the PTV, dose to the hottest 5% of the PTV below 115% of prescription, and hot spots restricted to within the PTV. Lung constraints are Lyman‐Kutcher‐Burman (LKB) normal tissue complication probability (NTCP) ≤25 ^(9)^ (approximately equivalent to total lung mean dose below 20‐21 Gy) and V20Gy<37%–40%. Other constraints and the sources from which they are derived are provided in Table 1.^10^, ^11^, ^12^, ^13^, ^14^ The attending physician either approved deviations from these or decreased the prescribed number of fractions until constraints were satisfied. Since two most serious complications are radiation pneumonitis and radiation myelitis, our two most strictly followed normal tissue limits are NTCP ≤25 and maximum spinal cord dose ≤50Gy.

**Table 1:** Institutional planning criteria for hemithoracic IMRT for MPM. Quantities in parentheses are the highest permitted without special physician consideration.

*Target Criteria*				*Note*
PTV	D95%	≥	94%	
	V95%	≥	94%	
	D05%	≤	115%	
	Hot spots are inside the PTV			
*Normal tissue Criteria*				
Lung				
Total Lungs	Mean dose	≥	21Gy	^(10)^
	V20 Gy	≥	37%	^(11)^
	NTCP	≤	25%	^(12)^
Cord	Maximum point dose	≤	50Gy	^(10)^
Bowel (upper abdomen)	Maximum point dose	≤	55Gy	
	D05%	≤	50Gy	
Heart	V30 Gy	≤	50%	^(13)^
	Mean Dose	≤	30Gy	^(13)^
Kidney	V18Gy	≤	33%	
Liver	Mean Dose	≤	30Gy	^(10)^
	V30 Gy	≤	50%	
Stomach	Mean Dose	≤	30Gy	Stomach not PTV
Esophagus	Mean Dose	≤	34Gy	^(10)^
	V60Gy	≤	17%	^(14)^

### B. Design and statistical methods

Five volumes were recorded for each patient: the ipsilateral lung (IL), contralateral lung (CL), total lung, PTV, and ipsilateral normal lung (ipsilateral lung volume excluding overlap with PTV). These volumes and their ratios were all obtainable from the planning CT scan without requiring a treatment plan. We analyzed these volumes and five volume ratios: contralateral/ipsilateral lung volumes (CIVR), contralateral lung volume/PTV (CPVR), ipsilateral lung volume/PTV (IPVR), ipsilateral normal lung volume/total lung volume (INTLVR), and ipsilateral normal lung/PTV volume (INLPVR). We also recorded the prescription dose and the mean doses to total lung (MLD), ipsilateral lung (MILD), and contralateral lung (MCLD) from each treatment plan.

Spearman's rank correlation was used to investigate the correlation between the prescription dose, the volume parameters, and the five volume ratios. Fisher's exact test was used to assess correlations between prescription dose and the categorical variables formed by above or below the median for the most significant volume ratio. We also used the Spearman's rank correlation to investigate correlation between MILD and the three ratios of lung volumes to PTV: CPVR, IPVR, and INLPVR, and between the prescription dose and the mean doses MILD and MCLD. Linear models were constructed for the most significant correlations.

Twenty‐three MPM patients with two lungs were treated with IMRT or volumetric‐modulated arc therapy (VMAT) in our clinic after 2012 with treatment plans that were designed without input from this study. Ten were planned in the in‐house planning system and 13 in Eclipse. The parameters which showed the most significant correlation in the training set were used to validate the models by comparing the predicted and attained prescription dose and MILD for these patients.

## III. RESULTS

### A. Plan characteristics

The prescription doses for the 56 patient training set were 50.4 Gy for 14 patients (25%), 48.6 Gy for 15 patients (27%), 46.8 Gy for 9 patients (16%), and 45.0 Gy or less for 18 patients (32%). Table 2 gives the patient characteristics and averages and ranges of the five examined volumes.

**Table 2:** Patient characteristics.

*Characteristic*		*Number of Patients (%)*
Surgery		
P/D or P		48 (86)
Nonoperative		8 (14)
Laterality		
Right		34 (61)
Left		22 (39)
Prescription Dose		
5040		14 (25)
4860		15 (27)
4680		9 (16)
≤4500		18 (32)
*Volume*	Average±SD	*Range*
PTV	2924.8.±908.6cc	1091.8 cc ‐ 6675.4 cc
Ipsilateral Lung	1208.2±425.4cc	477.1 cc ‐ 2340.7 cc
Ipsilateral Normal Lung	606.8±269.5cc	155.2 cc ‐ 1595.0 cc
Contralateral Lung	1677.3±469.8cc	763.1 cc ‐ 2756.3 cc
Total Lung	2911.0±790.3cc	1360.5 cc ‐ 5030.1 cc

The medians and ranges for the volume ratios were 1.33 for CIVR (0.83‐3.70), 0.58 for CPVR (0.31‐1.09), 0.43 for IPVR (0.08‐0.89), 0.22 for INTLVR (0.05‐0.42), and 0.21 for INLPVR (0.05‐0.60).

The average mean dose, standard deviation and range over all patients for the ipsilateral lung was 40.1±4.4 Gy (33.5 Gy–49.9 Gy), for the contralateral lung was 5.7±1.9 Gy (1.8 Gy–10.8 Gy), and for the total lung was 19.9±1.0 Gy (16.2 Gy–20.7 Gy).

### B. Correlations

**Figure 2:**
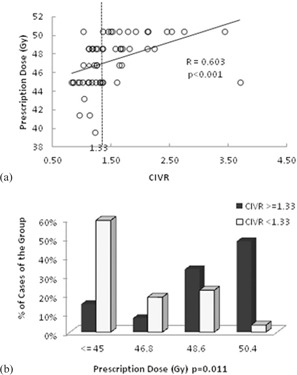
Correlation between CIVR and prescription dose; each circle is a patient: (a) linear regression fit for ratio of CIVR) versus prescription dose (slope=1.98,intercept=44.3Gy); (b) distribution of patients above and below the median CIVR among the different prescription levels. Lower prescriptions of two cases with high CIVR (1.684 and 3.699 were limited by other OARs' dose.

Table 3 shows the correlations of the variables discussed below with achieved prescription dose. Correlation with CIVR was most significant, with R=0.603(p<0.001). Figure 2(a) shows the linear regression line for this correlation. Prescription dose had a weaker correlation with IPVR and with the ipsilateral lung volume (IL) than CIVR; these two correlations were negative. Correlation with the other ratios and volumes tested did not reach statistical significance and hence are not mentioned in Table 3. To further investigate the correlation of CIVR and prescription dose, we divided patients into two groups, split at the median CIVR value, 1.33. The median split was significant (p<0.001) by Fisher's exact test. Figure 2(b) shows the distribution of these two CIVR groups among the different prescription doses. For CIVR≥1.33,79% of cases received 48.6 Gy or 50.4 Gy (46% received 50.4 Gy). For CIVR<1.33, only 25% of cases received 48.6‐50.4 Gy (4% received 50.4 Gy). The sensitivity and specificity of median CIVR for predicting prescription dose above 48.6 Gy were 0.759 and 0.778, respectively. For predicting prescription dose of 50.4 Gy the sensitivity and specificity of the median CIVR were 0.929 and 0.643, respectively.

**Table 3:** Spearman's correlation coefficient (R) and p value (p) for prescription dose and significantly correlated variables.

	*IL*	*CIVR*	*IPVR*	*MILD*	*MCLD*
R	−0.445	0.603	−0.351	0.554	0.240
p	0.001	<0.001	0.008	<0.001	0.075

**Figure 3:**
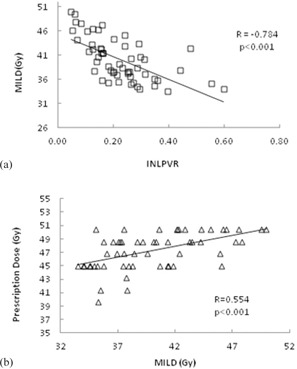
The correlation and linear regression fit (a) for MILD versus INLPVR(slope=‐23.6;intercept=45.4Gy); and (b) for prescription dose versus MILD(slope=0.33;intercept=34.1Gy).

Mean ipsilateral lung dose (MILD), which we routinely use as a constraint in the optimization process, was negatively correlated with the volume ratios IPVR(R=‐0.658; p<0.001) and INLPVR(R=‐0.784; p<0.001). Because these two volume ratios were also strongly correlated with each other (R=0.870; p<0.001) we created a linear model (Fig. 3(a)) to predict the lowest achievable MILD from INLPVR which had the higher correlation with MILD. MILD was also significantly correlated with prescription dose (Table 3 and Fig. 3(b)) although INLPVR was not (R=‐0.113; p=0.406). Use of INLPVR for an anatomy‐based estimate of MILD to use at an intermediate stage of optimization can help to more efficiently drive the optimization toward the highest achievable prescription dose consistent with the constraints.

### C. Validation patients

**Figure 4:**
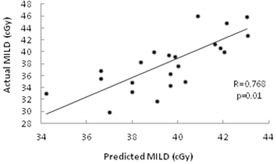
Validation of prediction model in 23 test cases: actual MILD vs. MILD predicted by the previously derived linear relation with measured INLPVR.

According to the predictive model, if CIVR≥1.33, the prescription dose that satisfies the lung OAR constraints should be 48.6 Gy or higher, while if CIVR is <1.33, the prescription dose should be less than 48.6 Gy. We applied this model to the next 23 patients who were consecutively treated in our department. The median CIVR of the validation patients was 1.27. Nine patients had CIVR≥1.33 (range 1.33‐2.58) and prescription doses of eight of these were ≥48.6Gy while one was 46.8 Gy due to the heart constraint. Fourteen patients had CIVR<1.33 (range 0.67‐1.32) and prescription doses of eleven of these were ≤46.8Gy while three were ≥48.6Gy; these had CIVR between 1.27 and 1.32. Although the training set median CIVR was lower, the training set median split of CIVR remained highly significant for the validation set (p<0.003) by Fisher's exact test. The MILD for these patients' plans was also well predicted by the linear relation with INLPVR (Fig. 3(b)) as shown in Fig. 4 (R=0.768; p=0.01).

## IV. DISCUSSION

Adjuvant hemithoracic pleural IMRT is a promising technique to treat MPM patients with two intact lungs.^6^, ^15^ Because plan optimization is complex and lung dosimetric constraints often limit the achieved prescription dose, we developed a simple anatomy‐based method to estimate a realistic prescription goal before starting optimization, thus providing both physician and planner with a “heads up” regarding the difficulty of the case. This is the first study to evaluate the relationship between patient anatomy and achievable prescribed dose in IMRT plans for these patients.

Several studies have used a combination of the geometry of patient anatomy and their clinical treatment plans to estimate achievable dosimetric limits before starting to plan. Hunt et al.^(16)^ analyzed delivered IMRT plans of 51 head‐and‐neck patients and showed that the ability to limit the parotid gland mean dose to the desired 26 Gy could be predicted by the percent of volume overlap of the gland with the PTV. Moore et al.^(17)^ analyzed the OAR doses delivered by 42 clinical IMRT head‐and‐neck or prostate cancer treatment plans and showed that the ratio of a minimum OAR dose to the prescription dose could be predicted by the fraction of the OAR volume overlapping the PTV. Wu et al.^(18)^ first focused on the overlap of parotid glands with PTV in a database of 32 head‐and‐neck patients and identified 13 patients where the parotid DVH could be improved by replanning. Subsequently, this group generated overlap volume histograms (OVH) for other organs and used this methodology to generate starting optimization objectives to improve the efficiency of IMRT optimization for head‐and‐neck patients.^(19)^

Our study investigated five volumes that are easily measured in the planning scan prior to optimization and five ratios that could limit the attainable prescription dose subject to our lung constraints. Other volume ratios were investigated but were found to provide redundant information. For example, the contralateral to total lung volume ratio and the CIVR are highly correlated (R=0.957) and provide essentially the same predictive information (data not shown).

The strongest correlation was with the ratio of contralateral to ipsilateral lung volumes (CIVR). For the 23 validation patients, CIVR was confirmed as an excellent predictor of the attained prescription. Also, for both sets of patients, INLPVR predicted MILD, which our planners often use as an optimization constraint. Although these correlations were derived from a training set planned in the in‐house planning system, they remained predictive for patients planned in Eclipse.

With any dataset of “real‐world” plans, anatomic variations and inherently associated variations in CIVR values are expected and unavoidable. CIVR is not an absolute determinant of prescription dose, but rather one source of guidance as to how realistic the planning goal might be for a specific patient. Factors such as protection of other OARs, planner skill, or allotted time and patient‐specific physician's goals may limit the prescription dose despite high CIVR. Anatomical factors, such as a PTV that crosses the mediastinal midline, may lead to higher contralateral and total lung mean doses than anticipated. Similarly, patients with lower CIVRs may have other anatomic features that allow higher doses. Especially when CIVR is slightly below the chosen cutpoint, a persistent and skilful planner may achieve the prescription goal.

A limitation of this study is that the input comes from a particular IMRT technique (Fig. 1) and set of plan evaluation metrics, an experienced group of treatment planners, and two particular treatment planning systems' dose calculation and optimization algorithms. While we expect that CIVR would remain a significant predictor of achievable prescription dose for IMRT MPM cases planned on different treatment planning systems and with different techniques, details of the correlation, such as those shown in Fig. 2, and the chosen predictive value, could well be different. No geometric predictor would be needed if the desired prescription dose is much lower than ours. Therefore, planners in other departments should evaluate the applicability of this geometric predictor to their cases before putting it into use.

## V. CONCLUSIONS

For MPM patients with two intact lungs, a higher ratio of contralateral to ipsilateral lung volume (CIVR) predicts the ability of an IMRT plan to achieve a prescription dose of at least 48.6 Gy while maintaining an LKB model lung NTCP ≤25. For patients with high CIVR, other normal tissue constraints may be more dose‐limiting than the lungs. However, if the contralateral lung is small and CIVR is low, the prescription for the resulting plan will likely be lower than what was originally desired. Whether other anatomical factors are also significantly associated with the highest achievable prescription dose requires further investigation.

## COPYRIGHT

This work is licensed under a Creative Commons Attribution 4.0 International License.

## References

[1] Ahamad A , Stevens CW , Smythe WR , et al. Intensity‐modulated radiation therapy: a novel approach to the management of malignant pleural mesothelioma. Int J Radiat Oncol Biol Phys. 2003;55(3):768–75. 12573764 10.1016/s0360-3016(02)04151-2

[2] Rimner A and Rosenzweig KE . Novel radiation therapy approaches in malignant pleural mesothelioma. Ann Cardiothorac Surg. 2012;1(4):457–61. 23977536 10.3978/j.issn.2225-319X.2012.10.07 PMC3741784

[3] Gupta V , Mychalczak B , Krug L , et al. Hemithoracic radiation therapy after pleurectomy/decortication for malignant pleural mesothelioma. Int J Radiat Oncol Biol Phys. 2005;63(4):1045–52. 16054774 10.1016/j.ijrobp.2005.03.041

[4] Allen AM , Czerminska M , Jänne PA , et al. Fatal pneumonitis associated with intensity‐modulated radiation therapy for mesothelioma. Int J Radiat Oncol Biol Phys. 2006;65(3):640–45. 16751058 10.1016/j.ijrobp.2006.03.012

[5] Rice DC , Smythe WR , Liao Z , et al. Dose‐dependent pulmonary toxicity after postoperative intensity‐modulated radiotherapy for malignant pleural mesothelioma. Int J Radiat Oncol Biol Phys. 2007;69(2):350–57. 17467922 10.1016/j.ijrobp.2007.03.011

[6] Rosenzweig KE , Zauderer MG , Laser B , et al. Pleural intensity‐modulated radiotherapy for malignant pleural mesothelioma. Int J Radiat Oncol Biol Phys. 2012;83(4):1278–83. 22607910 10.1016/j.ijrobp.2011.09.027 PMC4359620

[7] Mohan R , Barest G , Brewster LJ , et al. A comprehensive three‐dimensional radiation treatment planning system. Int J Radiat Oncol Biol Phys. 1988;15(2):481–95. 3403328 10.1016/s0360-3016(98)90033-5

[8] Spirou S , Chui C . Generation of arbitrary intensity profiles by dynamic jaws or multileaf collimators. Med Phys. 1994;21(7):1031–41. 7968833 10.1118/1.597345

[9] Burman C , Kutcher GJ , Emami B , Goitein M . Fitting of normal tissue tolerance data to an analytic function. Int J Radiat Oncol Biol Phys. 1991;21(1):121–35. 10.1016/0360-3016(91)90172-z 2032883

[10] Marks LB , Yorke ED , Jackson A , et al. Use of normal tissue complication probability models in the clinic. Int J Radiat Oncol Biol Phys. 2010;76(3 Suppl):S10–S19. 20171502 10.1016/j.ijrobp.2009.07.1754 PMC4041542

[11] Graham MV , Purdy JA , Emami B , et al. Clinical dose‐volume histogram analysis for pneumonitis after 3D treatment for non‐small cell lung cancer (NSCLC). Int J Radiat Oncol Biol Phys. 1999;45(2):323–29. 10487552 10.1016/s0360-3016(99)00183-2

[12] Rosenzweig KE , Fox JL , Yorke E , et al, Results of a phase I dose‐escalation study using three‐dimensional conformal radiotherapy in the treatment of inoperable nonsmall cell lung carcinoma. Cancer. 2005;103(10):2118–27. 15830346 10.1002/cncr.21007

[13] NCI Community Oncology Research Program Protocol Summary. RTOG 1106: Randomized phase II trial of individualized adaptive radiotherapy using during‐treatment FDG‐PET/CT and modern technology in locally advanced non‐small cell lung cancer (NSCLC). Available at: http://www.kccop.org/cancer‐trials/lung/index.cgi/summary?tID=64

[14] Palma DA , Senan S , Oberije C , et al. Predicting Esophagitis after chemoradiation therapy for non‐small cell lung cancer: an individual patient data meta‐analysis. Int J Radiat Oncol Biol Phys. 2013;87(4):690–96. 24035329 10.1016/j.ijrobp.2013.07.029

[15] Minatel E , Trovo M , Polesei J , et al. Radical pleurectomy/decortication followed by high dose of radiation therapy for malignant pleural mesothelioma. Final results with long‐term follow‐up. Lung Cancer. 2014;83(1):78–82. 24216141 10.1016/j.lungcan.2013.10.013

[16] Hunt MA , Jackson A , Narayana A , Lee N . Geometric factors influencing dosimetric sparing of the parotid glands using IMRT. Int J Radiat Oncol Biol Phys. 2006;66(1):296–304. 16904529 10.1016/j.ijrobp.2006.05.028

[17] Moore KL , Brame RS , Low DA , Mutic S . Experience‐based quality control of clinical intensity‐modulated radiotherapy planning. Int J Radiat Oncol Biol Phys. 2011;81(2):545–51. 21277097 10.1016/j.ijrobp.2010.11.030

[18] Wu B , Ricchetti F , Sanguineti G , et al. Patient geometry‐driven information retrieval for IMRT treatment planning quality control. Med Phys. 2009;36(12):5497–505. 20095262 10.1118/1.3253464

[19] Wu B , Ricchetti F , Sanguineti G , et al. Data‐driven approach to generating achievable dose‐volume histogram objectives in intensity‐modulated radiotherapy planning. Int J Radiat Oncol Biol Phys. 2011;79(4):1241–47. 20800382 10.1016/j.ijrobp.2010.05.026

